# Why Reaching Zero-Dose Children Holds the Key to Achieving the Sustainable Development Goals

**DOI:** 10.3390/vaccines11040781

**Published:** 2023-03-31

**Authors:** Dan Hogan, Anuradha Gupta

**Affiliations:** 1 Gavi, The Vaccine Alliance, 1218 Le Grand-Saconnex, Switzerland; 2 Sabin Vaccine Institute, Washington, DC 20037, USA

**Keywords:** zero-dose children, underimmunized children, equity, multiple deprivation, Immunization Agenda 2030

## Abstract

Immunization has one of the highest coverage levels of any health intervention, yet there remain zero-dose children, defined as those who do not receive any routine immunizations. There were 18.2 million zero-dose children in 2021, and as they accounted for over 70% of all underimmunized children, reaching zero-dose children will be essential to meeting ambitious immunization coverage targets by 2030. While certain geographic locations, such as urban slum, remote rural, and conflict-affected settings, may place a child at higher risk of being zero-dose, zero-dose children are found in many places, and understanding the social, political, and economic barriers they face will be key to designing sustainable programs to reach them. This includes gender-related barriers to immunization and, in some countries, barriers related to ethnicity and religion, as well as the unique challenges associated with reaching nomadic, displaced, or migrant populations. Zero-dose children and their families face multiple deprivations related to wealth, education, water and sanitation, nutrition, and access to other health services, and they account for one-third of all child deaths in low- and middle-income countries. Reaching zero-dose children and missed communities is therefore critical to achieving the Sustainable Development Goals commitment to “leave no one behind”.

## 1. Introduction

The Sustainable Development Goals (SDGs) place great emphasis on equity with a shared commitment to “leave no one behind”. However, surveying the SDG indicators and related targets reveals that they place their measurement focus on national averages rather than disadvantaged or marginalized populations. Reducing child and maternal mortality, ending the epidemics of AIDS, tuberculosis, malaria, and neglected tropical diseases, and raising the coverage of essential services will all require health systems to reach disadvantaged and hard-to-reach populations suffering from a disproportionately high burden of morbidity and mortality. Therefore, direct measures of, and focus on, communities left behind are critical for the design of equitable health programs and for the success of the SDGs.

Immunization has one of the highest coverage levels of any health intervention [1] and therefore can be a pathfinder for other services and interventions. Immunization also provides substantial health and economic benefits, with an estimated 50 million future deaths averted through immunization activities in 2000–2019 [2], and USD 26 in economic benefits through averted costs of illness for every USD 1 spent on immunization between 2011 and 2020 [3] in low- and middle-income countries (LMICs). However, globally, in 2021, over 18 million infants failed to receive even the first dose of the basic diphtheria-tetanus-pertussis-containing vaccine (DTP1). These zero-dose children are markers of missed communities facing multiple deprivations, with two-thirds of zero-dose children living below the international poverty line of USD 1.90 per day [4]. Reaching them with immunization services can connect them and their families to the health system and other services, and all the health, economic, and social benefits that come with that. This includes poverty reduction (SDG1), better nutrition (SDG2), improved educational outcomes (SDG4), and reductions in inequalities (SDG10). In this Perspective, we explain how a focus on zero-dose children offers a pragmatic entry point for designing and reinvigorating programs and systems to achieve immunization commitments made by countries through the Immunization Agenda 2030 (IA2030) [5], including a 50% reduction in zero-dose children by 2030, and more broadly, to ensure that the aspiration of SDGs to leave no one behind is achieved.

## 2. Definition of a Zero-Dose Child

The term “zero-dose child” refers to a child who has failed to receive any routine immunizations. For monitoring purposes, it is a measure of whether a surviving infant has received at least one dose of the DTP vaccine. The focus on routine immunization as opposed to doses received through immunization campaigns is intentional, as the indicator aims to measure the reliable reach of immunization services, extended sustainably to reach all communities to achieve universal coverage. The choice of the lack of DTP as the indicator is a pragmatic one. While surveys can measure whether a child has received no doses of any vaccines, most administrative data systems report aggregated data that do not allow for the joint measurement of the receipt (or lack of receipt) of different vaccines. Vaccines other than DTP could also be considered as proxy indicators. DTP is preferred for global IA2030 monitoring, as the measurement of measles and polio vaccine coverage through household surveys may contain a mix of routine and campaign-delivered doses, and the BCG vaccine is not in every country’s national schedule and is delivered through diverse platforms. At the population level, low coverage of the DTP, BCG, MCV, or polio vaccine tends to be highly correlated with the prevalence of children who have received no immunizations, and therefore, the choice of the metric is less important than the programmatic aim of identifying and reaching missed communities with dependable immunization services [6].

## 3. Reaching Zero-Dose Children to Accelerate Equitable Immunization

National immunization programs have made impressive gains in the past two decades, as many children, including those in low- and middle-income countries, are now protected against the leading causes of pneumonia, diarrhea, meningitis, and liver disease. Breadth of protection, defined by WHO to be the average coverage across 11 vaccines, doubled from 34% in 2000 to 68% in 2021 [7], meaning an increased number of children in the world are now protected against an array of vaccine-preventable diseases. However, while many life-saving vaccines have been added to national immunization schedules, some children continue to be deprived of the benefits of even the most basic vaccines in almost all countries. 

**Figure 1:**
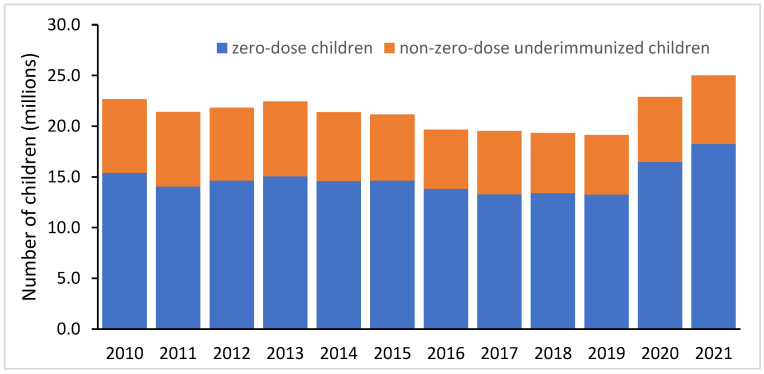
Annual number of zero-dose children and non-zero-dose underimmunized children globally, 2010–2021. Data source: WHO/UNICEF Estimates of National Immunization Coverage (WUENIC), July 2022 [8].

In 2021, there were 25 million underimmunized infants worldwide, as measured by the lack of three doses of the DTP-containing vaccine (DTP3) (Figure 1), which is the standard measure of the strength of routine immunization systems [8]. However, of these 25 million children, 18.2 m (73%) were zero-dose children, highlighting how essential it will be to reach zero-dose children to improve routine immunization coverage. The importance of focusing on zero-dose children is apparent when considering trends over the past decade. Coverage with three doses of the DTP-containing vaccine (DTP3) rose by 11 percentage points between 2000 (72%) and 2010 (83%) but then by only 3 percentage points between 2010 and 2019 (86%) [8]. The modest increase in DTP3 coverage in the decade prior to the COVID-19 pandemic was largely driven by a reduction in the percentage of children who had received their first dose of DTP but failed to receive their second or third doses of DTP; i.e., DTP drop-out decreased by about one-third (6.7% to 4.4%). In comparison, the coverage of DTP1 increased by only 1 percentage point between 2010 (89%) and 2019 (90%), meaning 1 in 10 children were zero-dose children prior to the pandemic [8]. Increasing DTP3 coverage will therefore be dependent on reaching zero-dose children and ensuring they are fully immunized.

There is also evidence that reaching a zero-dose child may catalyze a cascade of further vaccinations. In an analysis of household survey data from 92 LMICs considering four basic vaccines, most children had either received no doses of any vaccines or received doses of three or more different vaccines [6]. This finding suggests that reaching zero-dose children should be a major focus of immunization programs seeking to increase full immunization coverage, as children who receive one dose almost always move on to receive several other vaccinations.

## 4. Where Are Zero-Dose Children?

Most zero-dose children live in low- and lower-middle-income countries, accounting for 87% of the global total of 18 million [8]. In 2021, six large-population countries, namely, India (2.7 m), Nigeria (2.2 m), Indonesia (1.1 m), Ethiopia (1.1 m), Philippines (1 m), and the Democratic Republic of the Congo (0.7 m), accounted for half of all zero-dose children. There are also smaller countries that have chronically low coverage and a very high proportion of zero-dose children who are zero-dose even without COVID-related disruptions, for example, Papua New Guinea (56%), South Sudan (49%), Somalia (48%), and Central African Republic (46%) as of 2019 [8]. All of these countries face fragility and conflict, which lead to weaker and less predictable immunization delivery. 

High rates of zero-dose children in fragile and conflict settings also play out at the subnational level across countries. An analysis that combined conflict data from the Armed Conflict Location & Event Data Project (ACLED) with subnational coverage estimates from the Institute for Health Metrics and Evaluation (IHME) found that nearly 20% of zero-dose children in 99 LMICs live in conflict-affected settings [9]. The same analysis also concluded that roughly 40% of zero-dose children live in settings highlighted by the Equity Reference Group on Immunization (ERG), namely, urban, remote rural, and conflict-affected settings, with the remaining living in non-urban rural settings. In related work, Utazi et al. found high rates of zero-dose children in conflict-affected and remote rural regions, which are common in parts of the Sahel and the Horn of Africa [10].

More data are needed to quantify the sizes of zero-dose populations in urban slums at the global level, as they are often not captured by household surveys and are geographically too small for vaccine coverage levels to be estimated with geostatistical models. Work that has been conducted suggests that children living in slums may have better access to services than those in rural areas but still face large inequalities compared to wealthier urban households [10,11]. 

Overall, zero-dose children live in every country in the world. In many countries, the prevalence of zero-dose children can vary substantially across subnational areas. For example, geospatial modeling of subnational DTP1 coverage in Africa found that Angola, Chad, the Democratic Republic of the Congo, Ethiopia, Kenya, Mali, and Nigeria all had mean disparities in DTP1 coverage of 50% or more at the second administrative level [12]. The geographic targeting of resources to support the expansion of routine immunization services to reach missed communities is therefore critical. However, while geographic information can help target resources to reach chronically missed children, in many countries, other factors may be more important than just the geographic setting in determining why children are unvaccinated [10]. As zero-dose children often face multiple barriers to immunization, understanding the social, political, and economic contexts of zero-dose children and their families is key for program design.

## 5. Who Are Zero-Dose Children and What Barriers Do They Face?

Recent empirical studies by the International Center for Equity in Health and others have confirmed what most public health practitioners have long known: zero-dose children and their families face multiple barriers to obtaining immunization, and their presence in a community is often an indicator of compounded inequities. Moreover, stigma and discrimination are likely factors in determining whether a child benefits from vaccines. 

Gender-related barriers to immunization are a key driver of children missing out on vaccinations. Children with empowered mothers, as defined by the Survey-based Women’s emPowERment (SWPER) index, are much less likely to be zero-dose. In particular, in the domain of social independence, children whose mothers were measured to have low or medium levels of social independence were 3.3 times more likely to be zero-dose than children of mothers with high levels of social independence [13]. Although the analysis was not causal, the suggested effect sizes are enormous; theoretically, if barriers to immunization related to women’s empowerment could be overcome, there would be 4.7 million fewer zero-dose children globally. 

Consistent with the literature on inequalities in access to various health services [14,15], children from poorer households are more likely to be zero-dose than children from wealthier households. Unfortunately, there appears to have been little progress in reducing this gap over the past ten years, and the greatest absolute inequalities occur in the poorest countries, with low-income countries having a 14 percentage point difference in median zero-dose prevalence when comparing the poorest to wealthiest household quintiles [16]. Zero-dose children are often poor, with roughly two-thirds living below the poverty line of USD 1.90 per day [4]. 

Recent studies suggest that ethnicity and religion may contribute to disparities in immunization in some countries. In a study of 64 LIMCs, the median gap in the prevalence of zero-dose children between ethnic groups with the lowest vs. highest prevalence was 10 percentage points (pp), and gaps of 50 pp were observed in five countries [17]. Importantly, differences in zero-dose prevalence by ethnicity persisted even after controlling for wealth, maternal education, and area of residence, suggesting that other factors linked to ethnicity are key drivers of immunization inequalities in some countries. It is concerning that children from smaller ethnic groups in a country are more likely to be zero-dose than children in the dominant ethnic group [17]. The relationship between religion and immunization status appears to be significant in some countries but not consistently across countries [18]. In 27 of 66 countries studied, zero-dose prevalence varied by religious group, with children from the majority religion tending to be less likely to be zero-dose than children from minority religions, with the exception of countries where Muslims were the majority religion. 

One significant gap in the evidence base about zero-dose children is in understanding patterns among refugee, migrant, and nomadic populations. A recent review by the World Health Organization cited 26.4 million refugees in 2020 and 41.3 million internally displaced people due to violence and conflict in 2021, and while some of these populations experience lower immunization rates, it is context-specific with unclear patterns overall [19]. The size of nomadic, displaced, and migrant populations is dynamic and can be exacerbated by conflicts, climate shocks, food shortages, natural calamities, and loss of income. This in turn can increase the number of children who are missed by immunization services as well as household surveys designed to measure immunization coverage [20].

In addition to inequalities associated with accessing immunization, zero-dose children and their families face multiple deprivations related to health and development. Considering other child and maternal health services, zero-dose children and their mothers are roughly two times as likely to miss out on antenatal care and access to an institutional delivery, although interestingly, only about 20% less likely to access care for childhood illnesses or symptoms [21]. In an expanded analysis considering broader development indicators at the individual level, a lack of vaccination was strongly associated with lower access to improved water (prevalence ratio (PR) = 2.60) and sanitation (PR = 1.35), higher rates of childhood stunting (PR = 1.32), lower levels of maternal education (PR = 2.27), and lower levels of maternal demand for family planning satisfied with modern methods (PR = 1.42) [22]. Similar patterns were also observed in ecological analyses looking across countries and across subnational regions within countries, and a principal component analysis looking at these deprivation variables found that nearly all zero-dose children are in the highest deprivation quintile: i.e., if a zero-dose child is found, it is highly likely that they are facing multiple deprivations [22]. A geospatial analysis of time trends in zero-dose children in India from 1992 through 2016 found similar results, with zero-dose children more likely to be poor, have mothers with no education, suffer from severe stunting, and live in less developed states and districts [23].

## 6. What Is Needed to Sustainably Reach Zero-Dose Children?

The Immunization Agenda 2030 and the supporting Gavi 2021–2025 Strategy [24] have ambitious targets to reduce the number of zero-dose children by 25% by 2025 and 50% by 2030 as compared to 2019 levels. These targets are even more challenging following two years of backsliding in vaccination coverage during 2020 and 2021, resulting in an additional 5 million zero-dose children globally. Moreover, coverage disruptions due to COVID-related lockdowns in 2020 illustrated that gains in coverage among zero-dose children can be tenuous, as 95% of the increase in the number of underimmunized children in low- and lower-middle-income countries was due to an increase in zero-dose children [8]. Population growth presents another challenge. The 15 countries that had a zero-dose prevalence of 30% or more in 2021, accounting for 40% of all zero-dose children globally, are expected to see nearly a 10% increase in their birth cohorts in 2030 as compared to 2021 [8,25]. Thus, it will be important to design robust programs to sustainably reach zero-dose children to reach 2030 targets while avoiding a “one size fits all” approach.

The Identify-Reach-Monitor-Measure-Advocate (IRMMA) framework offers a way to develop strategies to reach zero-dose children and missed communities [26]. The IRMMA framework involves diving deeper into subnational- and community-level inequities and identifying where unvaccinated children live and what barriers to immunization they face. As the majority of zero-dose children tend to live in countries still developing their health information systems, data triangulation is often necessary, though imperfect. Tailored strategies appropriate for the local context then need to be designed and operationalized to overcome identified barriers. For example, strategies to sustainably reach zero-dose children with immunization services in urban slums would be different from those for nomadic populations or for children in cross-border settings. This will often require addressing gender barriers to immunization, and opportunities for integrated service delivery should be sought out to increase efficiency and sustainability and to take advantage of opportunities opened by vaccination. Supplemental immunization activities should also include the purposeful linking of newly reached zero-dose children back to the routine immunization system to ensure children go on to receive a full complement of vaccines. Such approaches also provide an opportunity to improve the data systems that enable the program’s ability to monitor and measure progress. Robust monitoring and measurement are critical for refining delivery approaches and advocating for pro-equity investments. Political will is necessary to initiate and sustain the program and should be secured with a purposeful and inclusive advocacy approach.

Several data and evidence gaps also warrant attention. These include the need for investment in improved demographic and immunization coverage data to enable the identification and monitoring of efforts to reach zero-dose children. To the extent that data from household surveys are used to quantify the distribution and characteristics of zero-dose children, in cases where survey sampling frames are outdated, the picture may be incomplete, and new methods relying on gridded population survey sampling warrant consideration [27]. New innovative methods to overcome barriers to immunization should also be tried, documented, and shared. This should include information on program costs. While there are estimates of average immunization delivery costs [28], data on the incremental costs associated with expanding the reach of immunization systems are very limited [29] but likely higher for hard-to-reach populations [30].

## 7. Impact of Reaching Zero-Dose Children and Missed Communities

**Figure 2:**
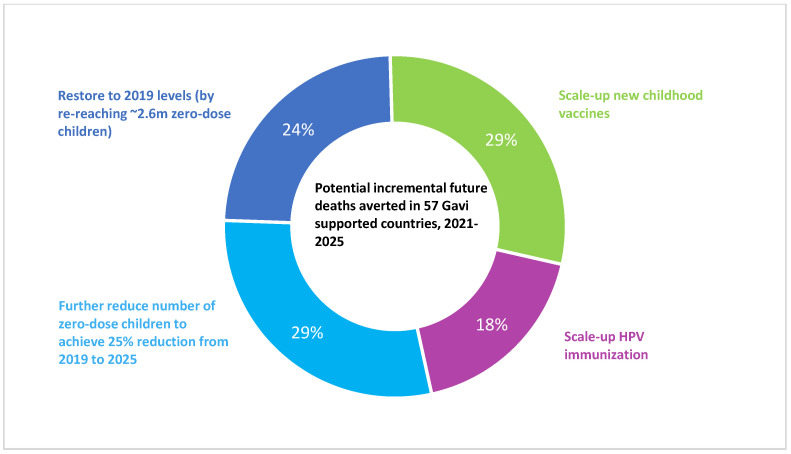
Potential incremental future deaths averted in 57 Gavi-supported countries through routine immunization, 2021–2025. Analysis based on Vaccine Impact Modeling Consortium impact ratios and immunization coverage as estimated in the WUENIC July 2021 release, ignoring the impact of maintaining coverage at 2020 levels and assuming Gavi 5.0 targets are met.

Reaching zero-dose children with a full complement of vaccines has the potential to substantially reduce child mortality, as nearly half of all vaccine-preventable deaths in LMICs occur among zero-dose children [26]. The impact of vaccination is potentially highest in zero-dose children, as they would otherwise be receiving no protection against vaccine-preventable diseases, be more susceptible to infection, and be the least likely to benefit from timely and high-quality treatment if they fall ill. In an analysis conducted by Gavi, the Vaccine Alliance based on data from the Vaccine Impact Modelling Consortium [2], immunizing zero-dose children would account for 53% of incremental impact in Gavi-supported countries through routine immunization between 2021 and 2025, with the remainder of the impact coming from scaling up new childhood vaccines among non-zero-dose children and HPV vaccination (Figure 2) [31]. A modeling study focused on 41 LMICs from 2021 to 2030 estimated that vaccination among the two poorest wealth quintiles would avert 1.2 to 3.8 times as many future deaths per person vaccinated as compared to vaccination in the two wealthiest quintiles [32]. The same study projected that vaccination would avert 24 million cases of medical impoverishment in 2021–2030, with more than 40% of the impact occurring within the poorest wealth quintile for many vaccines. Sustainably reaching communities currently missed by immunization would also help prevent future outbreaks, including the resurgence of measles and polio, and remove the need for repeated disease-specific supplemental immunization activities.

The potential impact of reaching zero-dose children and their communities goes beyond vaccine-preventable diseases. Nearly one-third of all-cause under-five child deaths in LMICs occur in households with a zero-dose child [33], so they must be a focus as countries strive for the SDG child mortality target of fewer than 25 under-five deaths per 1000 live births. Achieving the SDGs thus requires addressing the multiple deprivations faced by zero-dose children and missed communities through strengthened and integrated primary care, as well as improved water, sanitation, nutrition, and education.

## 8. Conclusions

Zero-dose children account for over 70% of underimmunized children and must be reached with sustainable immunization services to meet ambitious targets for 2030. Identifying and understanding zero-dose children and missed communities will be key for designing effective interventions to reach them, which will often require tailoring to the local context. As zero-dose children and their families face multiple deprivations, with a high burden of morbidity and mortality, the potential for impact is great if they can be reached. By doing so, countries would be taking a key step toward ensuring no one is left behind in the Sustainable Development Goal era.

## Data Availability

Data sharing not applicable.
